# Gastric resection with intrathoracic anastomosis in a hiatal hernia – A case report

**DOI:** 10.1016/j.ijscr.2022.107809

**Published:** 2022-11-29

**Authors:** Radek Vrba, Dušan Klos, Daniela Kürfurstová, Petr Špička

**Affiliations:** a Department of Surgery, Faculty of Medicine and Dentistry, Palacky University Olomouc, Hnevotinska 976/3, Olomouc 775 15, Czech Republic; b Department of Clinical and Molecular Pathology, Faculty of Medicine and Dentistry, Palacky University Olomouc, Hnevotinska 976/3, Olomouc 775 15, Czech Republic

**Keywords:** CT, computed tomography, BMI, body mass index, CT AG, computed tompography angiography, CRP, C-reactive protein, Leu, leucocytes, ICU, Intensive Care Unit, Mixed hiatal hernia, Gastric necrosis, Total gastrectomy

## Abstract

• Severe complication of hiatal hernia with gastric and esophageal necrosis
• Acute total gastrectomy with esophagojejunoanastomosis via right-sided thoracotomy
• Simultaneous resection of multiple jejunal diverticula present

## Abstract (cs)

**Introduction:**

In this case report we describe the surgical treatment of an incarcerated bulky mixed hiatal hernia with gastric necrosis, where the only treatment was extensive surgical resection of the stomach. The procedure included resection of the jejunum containing multiple jejunal diverticula.

**Case presentation:**

The patient was examined for acute shortness of breath, chest pain and recurrent vomiting. CT scan showed a bulky mixed hiatal hernia with gastrectasia. The patient was referred for surgical treatment due to acute symptoms. Perioperatively, a mixed hiatal hernia with gastric necrosis due to incarceration of the oral 2/3 of the stomach above the diaphragm was discovered. A total gastrectomy was performed, and due to ischemia of the esophagus, an esophagojejunoanastomosis via right-sided thoracotomy had to be performed. Multiple jejunal diverticula were a surprising perioperative finding and were resected. Postoperatively, the patient was admitted to the ICU. She was discharged to home care in stable condition, fully realimented.

**Clinical discussion:**

Patients with a bulky hiatal hernia are at risk of serious complications if it is incarcerated. Patients with acute symptomatology of the disease are indicated for acute surgical revision, and the surgical procedure is modified according to perioperative findings.

**Conclusion:**

Acute surgical treatment is indicated in patients with symptomatic hiatal hernias where there is a risk of serious complications. The type of surgery is modified according to the actual perioperative findings.

## 1 Introduction and importance

Hiatal hernias are divided into four basic types according to their morphology (axial, paraesophageal, mixed hiatal hernia and upside-down stomach) [1]. Paraesophageal hernias, bulky mixed hernias and especially upside-down stomach hernias are at risk of developing severe complications with the need for acute surgical intervention when they are acutely incarcerated or obstructed. In the reported case report, we describe an extensive resection surgery performed in our university hospital by laparotomy and thoracotomy for an incarcerated bulky mixed hiatal hernia. This paper has been reported in line with the SCARE 2020 criteria [2].

## 2 Case presentation

**Fig. 1:**
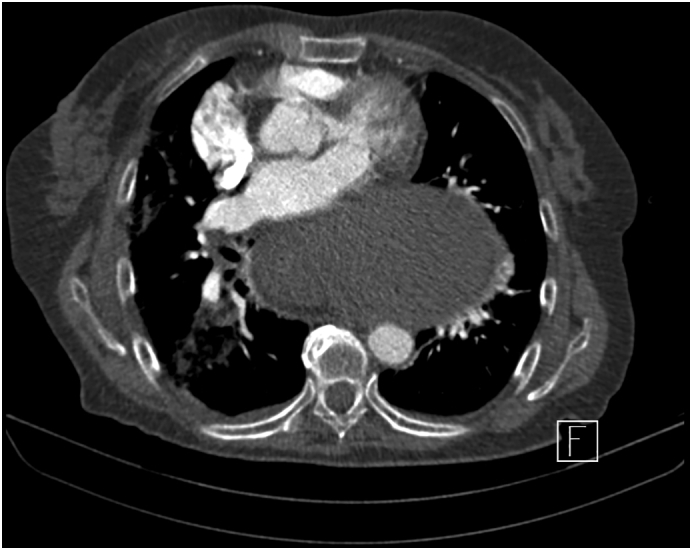
CT of mixed hiatal hernia - transverse section.

**Fig. 2:**
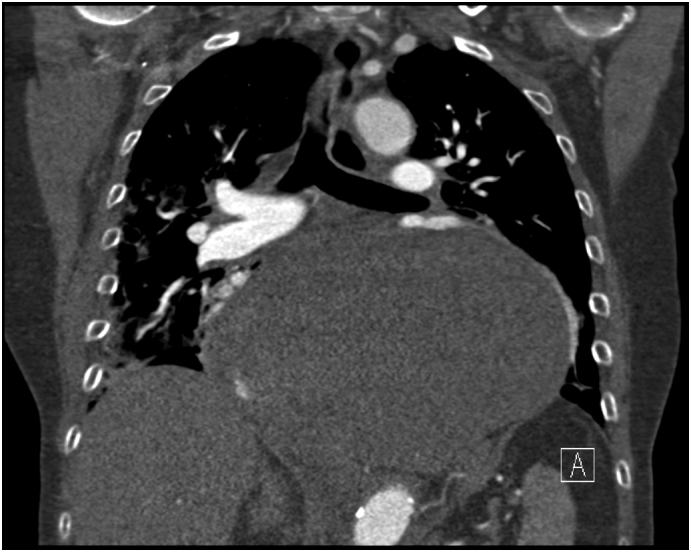
CT of mixed hiatal hernia - coronal section.

**Fig. 3:**
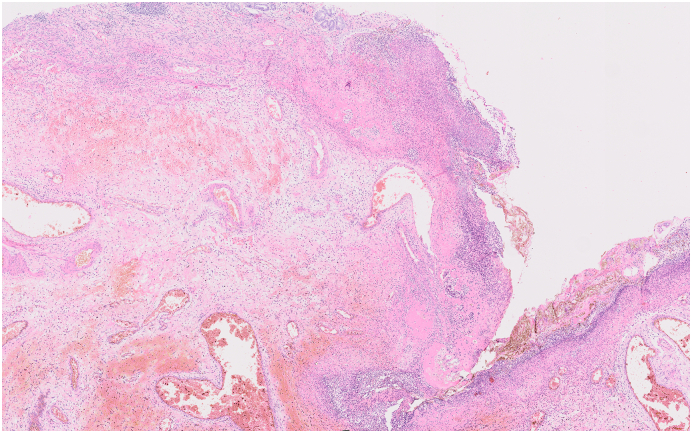
Histological examination of the necrosis of the resected stomach.

**Fig. 4:**
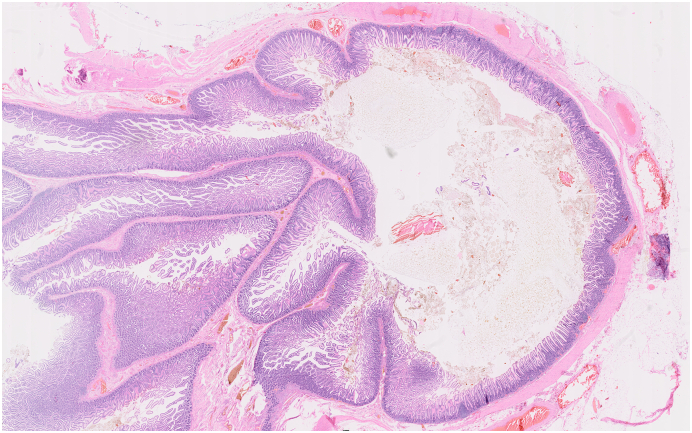
Histological examination of the resected jejunal diverticulum.

Patient I. M., female, born 1947, height 175 cm, weight 78 kg, BMI 25.4 was primarily admitted to the regional internal medicine department to rule out an acute coronary event. The patient suffered from attacks of acute shortness of breath, chest pain with repeated vomiting. She had a history of hypertension and hypoparathyroidism, treated pharmacologically; she had never undergone surgery. Cardiological examination ruled out a cardiological etiology. CT AG performed ruled out pulmonary embolism, but a bulky hiatal hernia with extensive gastrectasia was described (Figs. 1, 2). Laboratory values showed an elevation of inflammatory parameters CRP 73 mg/l, Leu 18.02 10 to 9/gl, lactate was within normal limits. A nasogastric tube was introduced and full parenteral, analgesic therapy was administered. For suspicion of incarceration of the mixed hiatal hernia, the patient was indicated for acute surgical revision. The surgery was performed by a surgeon specialized in upper GIT surgery with 30 years of experience. Perioperatively, a mixed hiatal hernia with the oral 2/3 of the stomach localized above the diaphragm with massive gastrectasia was discovered, the nasogastric tube was in place. Furthermore, on exploration of the abdominal cavity at a distance of 30 cm from the Treitz ligament, multiple (10–15) jejunal diverticula were found on the jejunum over a length of 40 cm. The stomach was repositioned back into the abdominal cavity, the area of the stomach above the diaphragm was incarcerated with signs of ischemic involvement with necrosis of the gastric wall. Due to the extent of involvement of virtually the entire stomach, the only possible treatment was to resect the stomach. A total gastrectomy was performed, and on revision of the distal esophagus, the esophagus was ischaemic and it was not possible to perform a safe esophagogastric anastomosis in the abdominal cavity. In this situation, resection of the jejunum with diverticula was performed and a Roux loop was constructed. The right-sided thoracotomy route was used to resect the distal esophagus with esophagojejunoanastomosis. Anastomosis was constructed with a circular 25 mm stapler. Two drains were introduced into the abdominal cavity and one drain into the thoracotomy. Surgery lasted 275 min, blood loss was 115 ml. In the postoperative period, the patient was hospitalized in the ICU, continued to be on artificial pulmonary ventilation, circulatory support with vasopressors, and was given a triple combination of antibiotics (Piperacillin/Tazobactam, Metronidazole, Fluconazole). On the first postoperative day, the patient had an attack of paroxysmal atrial fibrillation with rapid ventricular response, which was successfully treated pharmacologically. Abdominal and chest drains were successively extracted, and the patient was extubated on the eighth postoperative day. A control swallowing act was performed with favorable findings at the anastomosis. Laboratory values gradually normalized. The patient was transferred to a peripheral hospital for follow-up in stable condition on the 12th postoperative day, and was discharged to the care of a general practitioner on the 18th postoperative day. Histological examination described gastric necrosis affecting the mucosa, submucosa and muscular layer including subserosa (Fig. 3). Multiple diverticula were described in the small intestine (Fig. 4). Currently, the patient is clinically well 4 months after surgery, performance status 0. As far as her current condition is concerned, the patient is completely fine, feels well, has no major problems and is very grateful for saving her life.

## 3 Clinical discussion

In patients with hiatal hernias, patients with bulky paraesophageal, mixed hernias, and upside-down stomach are most often at risk for an acute course of the disease. Acute complications include gastric obstruction, acute bleeding, ischemia and gastric perforation. With gastric strangulation and ischaemia, patients are at risk of developing partial or complete gangrene of the stomach with subsequent perforation with the possibility of developing severe acute mediastinitis or peritonitis. In case of gastric obstruction with gastric contents, there is a risk of aspiration and development of pneumonia. The prevalence of acute conditions is reported in the literature to be 30.4 % [3]. Acute symptoms include acute epigastric pain, chest pain and recurrent vomiting in case of strangulation. Of the symptoms typical for acute incarceration of hiatal hernia, the patient in our case report presented with chest pain and acute recurrent vomiting. Patients usually report a history of reflux symptoms, but these may be absent in upside-down stomach. Paraesophageal hernia may also present with non-specific symptoms such as nausea, vomiting, hiccups, haematemesis, postprandial pain, dysphagia, dyspnoea [4]. Volvulus of the stomach may cause the so-called Borchardt's triad (non-productive gagging, epigastric pain and inability to insert a nasogastric tube) [5]. In the differential diagnosis, the disease may manifest as cardiac problems [6], [7]. The referred patient was primarily examined as a suspected acute cardiac event, but cardiological examination ruled out cardiac etiology and the CT scan correctly diagnosed incarcerated hiatal hernia. Diagnosis of the disease is established by CT scan of the chest and abdomen [8], with an elevation of inflammatory parameters in the laboratory results. Upside-down stomach is found in less than 5 % of hiatal hernias [3]. Other organs of the abdominal cavity (colon, omentum, spleen) may be part of the diaphragmatic hernia [9]. Untreated incarceration can be fatal with the development of serious complications (mediastinitis, peritonitis, septic shock) [7]. Surgical approach is recommended especially in symptomatic patients presenting with obstructive symptoms and volvulus [9]. Due to the acute symptomatology of the disease, the patient in our case report was immediately referred for surgical revision. The only treatment for acute incarcerated hiatal hernia is surgical, and the surgical procedure can be performed by conventional or minimally invasive approach. The principle of the operation is repositioning of the stomach back into the abdominal cavity, hiatoplasty and gastropexy to the abdominal wall. In the presence of reflux symptoms, an antireflux procedure in the form of 360 degree or partial fundoplication is indicated. The described surgical procedures are indicated in the absence of serious complications of the disease (perforation, gastric bleeding, gastric necrosis, mediastinitis, peritonitis). If any of these complications arise, the procedure is modified according to the perioperative findings with the aim of saving the patient's life [10], [11]. The high mortality of this disease is associated with its incidence in older age groups with the presence of severe internal comorbidities [12]. In our case, perioperative evidence of necrosis of virtually the entire stomach was found with the impossibility of resolving this serious condition by any other procedure but resection. On exploration of the abdominal cavity, a surprising finding was the presence of multiple jejunal diverticula. Jejunal diverticula are usually asymptomatic and are diagnosed only at surgical revision as an incidental finding or at operation for acute abdomen with pneumoperitoneum due to obstruction, bleeding or perforation [13]. Primarily, we indicated a total gastrectomy, but after gastric resection, an ischaemic abdominal esophagus was discovered and it was not possible to perform a viable anastomosis in the abdominal cavity. For this reason, we indicated an intrathoracic anastomosis via a right-sided thoracotomy and Roux loop. The jejunal diverticula were resected in their entirety during the construction of the Roux loop. Definitive histological examination of the stomach confirmed the perioperative finding of necrotic gastric wall in its entirety.

## 4 Conclusion

In case of acute herniated hiatal hernia, it is always a serious condition with the possibility of developing complications. In case of acute symptomatology of the disease, surgical therapy is the only curative option. The procedure can be performed using a minimally invasive or conventional approach, with the nature and extent of the procedure modified according to the actual perioperative findings. This complication is not often described in recent literature and the results of treatment are not yet encouraging. Therefore, our solution can be considered as a recommended procedure and can successfully treat or even save the patient's life.

## Provenance and peer review

Not commissioned, externally peer reviewed.

## Sources of funding

There are no sources of funding for our research.

## Ethical approval

Not required in our institution to publish Anonymous case reports.

## Consent

Written informed consent was obtained from the patient for publication of this case report and accompanying images. A copy of the written consent is available for review by the Editor-in-Chief of this journal on request.

## Research registration

No registry.

## Author contribution

Ass. prof. Radek Vrba, MD, Ph.D. – design of the study, collection on the data, final approval of the version to be submitted.

Ass. prof. JUDr. Dušan Klos, MD, Ph.D., MHA, LLM – design of the study, final approval of the version to be submitted.

Daniela Kurfürstová, MD, Ph.D. – collection on the histopathological data, approval of the version to be submitted.

Petr Špička, MD, Ph.D. – corresponding author, guarantor, revising the manuscript, final approval of the version to be submitted.

## Guarantor

Petr Špička, MD, Ph.D.

Ass. prof. Radek Vrba, MD, Ph.D.

## Declaration of competing interest

The author declared no conflict of interest.
